# Malaria incidence and entomological findings in an area targeted for a cluster-randomized controlled trial to prevent malaria in Ethiopia: results from a pilot study

**DOI:** 10.1186/s12936-016-1199-4

**Published:** 2016-03-08

**Authors:** Taye Gari, Oljira Kenea, Eskindir Loha, Wakgari Deressa, Alemayehu Hailu, Meshesha Balkew, Teshome Gebre-Michael, Bjarne Robberstad, Hans J. Overgaard, Bernt Lindtjørn

**Affiliations:** School of Public and Environmental Health, College of Medicine and Health Sciences, Hawassa University, Hawassa, Ethiopia; School of Public Health, College of Health Sciences, Addis Ababa University, Addis Ababa, Ethiopia; Aklilu Lemma Institute of Pathobiology, Addis Ababa University, Addis Ababa, Ethiopia; Centre for International Health, University of Bergen, Bergen, Norway; Norwegian University of Life Sciences, Ås, Norway; Institut de Recherche pour le Développement (IRD), Maladies Infectieuses et Vecteurs, Ecologie, Génétique, Evolution et Contrôle (MIVEGEC), Montpellier, France; Department of Entomology, Faculty of Agriculture, Kasetsart University, Bangkok, Thailand

**Keywords:** Malaria, Incidence, Indoor residual spraying, Long-lasting insecticidal nets, *Anopheles arabiensis*, Ethiopia

## Abstract

**Background:**

This study was part of the work to prepare for a cluster-randomized controlled trial to evaluate the effect of combining indoor residual spraying and long-lasting insecticidal nets on malaria incidence. A pilot study was done to estimate the variations of malaria incidence among villages, combined with entomological collections and an assessment of susceptibility to insecticides in malaria vectors.

**Methods:**

A cohort of 5309 residents from four *kebeles* (the lowest government administrative unit) in 996 households was followed from August to December 2013 in south-central Ethiopia. Blood samples were collected by a finger prick for a microscopic examination of malaria infections. A multilevel mixed effect model was applied to measure the predictors of malaria episode. Adult mosquitoes were collected using light traps set indoors close to a sleeping person, pyrethrum spray sheet catches and artificial outdoor pit shelters. Enzyme-linked immunosorbent assays were used to detect the sources of mosquito blood meals, while mosquito longevity was estimated based on parity. The World Health Organization’s tube bioassay test was used to assess the insecticide susceptibility status of malaria vectors to pyrethroids and carbamates.

**Results:**

The average incidence of malaria episode was 4.6 per 10,000 person weeks of observation. The age group from 5 to 14 years (IRR = 2.7; 95 % CI 1.1–6.6) and *kebeles* near a lake or river (IRR = 14.2, 95 % CI 3.1–64) were significantly associated with malaria episode. Only 271 (27.3 %) of the households owned insecticide-treated nets. Of 232 adult *Anopheles* mosquitoes collected, *Anopheles arabiensis* (71.1 %) was the predominant species. The average longevity of *An. arabiensis* was 14 days (range: 7–25 human blood index days). The overall human blood index (0.69) for *An. arabiensis* was higher than the bovine blood index (0.38). Statistically significant differences in *Anopheline* mosquitoes abundance were observed between the *kebeles* (P = 0.001). *Anopheles arabiensis* was susceptible to propoxur, but resistant to pyrethroids. However, *An. pharoensis* was susceptible to all pyrethroids and carbamates tested.

**Conclusions:**

This study showed a high variation in malaria incidence and *Anopheles* between *kebeles.* The observed susceptibility of the malaria vectors to propoxur warrants using this insecticide for indoor residual spraying, and the results from this study will be used as a baseline for the trial.

## Background

Long-lasting insecticidal nets (LLINs) and indoor residual spraying (IRS) are the two main malaria vector control tools available today [1]. A worldwide coordinated effort achieved a 47 % reduction of deaths from malaria since 2000 by using the existing interventions. However, in its 2014 report, the World Health Organization (WHO) indicated that malaria continues to be a major cause of morbidity with 198 million cases globally, of which 82 % were from Africa [2].

In Ethiopia, malaria is one of the major public health problems. The dominant malaria parasites are *Plasmodium falciparum* (77 % of all reported malaria cases) and *P. vivax* (33 %) [3], with *Anopheles arabiensis* being the main vector [4]. The transmission is seasonal, and determined by altitude, rainfall and temperature [5, 6], in addition to local epidemiology. The first malaria prevention and control strategies in Ethiopia began as pilot projects in the 1950s [7], and are now integrated into the national basic health services [8]. Early diagnosis and treatment of cases, LLINs and IRS are currently the main malaria prevention and control tools [9]. Despite the successes [2] and efforts made thus far, malaria in Ethiopia remains the main cause of morbidity (3,331,599 confirmed cases) and hospital admissions (59,370 cases) in 2012/13 [10].

In Ethiopia, IRS and LLINs are commonly used, both separately or in combination, in the same households [9]. The individual effect of both IRS and LLINs on malaria incidence is quite well documented, but evidence is scarce and contradictory as to the effect of combining them [11, 12]. A cluster-randomized controlled trial examining the utility of each intervention, as well as their combined potential to prevent malaria transmission, will be carried out in Ethiopia to help provide useful information for policymakers and health service managers [13]. In order to effectively implement the trial, measuring baseline data related to the existing vector species composition, density, infectivity, insecticide susceptibility status and malaria incidence were of paramount importance. Therefore, a pilot study was carried out in 2013 to collect epidemiological and entomological data from the trial area to assess variation in malaria episodes and local vector populations within and among study *gares* (a “*gare*” is a local name for village), and to determine insecticide susceptibility status.

## Methods

### Study area

**Fig. 1:**
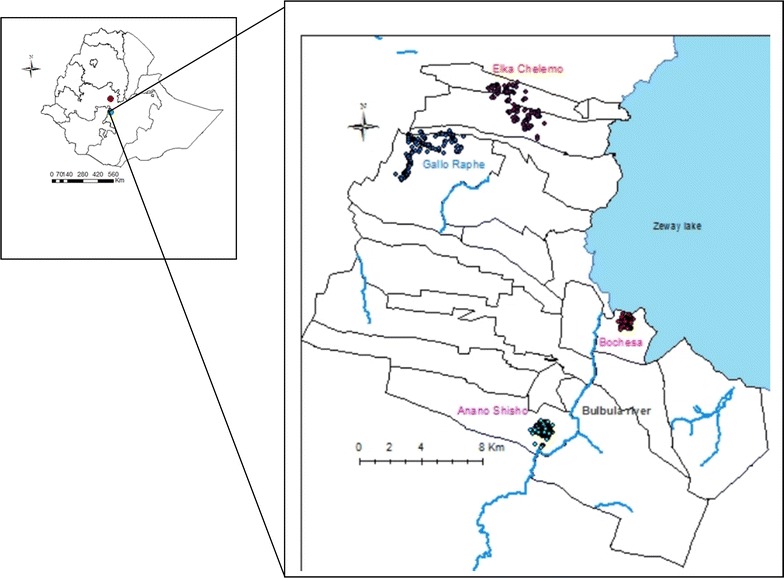
Geographic location of selected *kebeles*, Adami Tullu district, Ethiopia

This pilot study was conducted in the Adami Tullu part of the Adami Tullu-Judo-Kombolcha district (hereafter referred to as the Adami Tullu district) located 160 km south of Addis Ababa, the capital of Ethiopia, and described in detail elsewhere [13]. Briefly, the size of the district is 1403 square kilometres [14] and administratively divided into 48 *kebeles* (the lowest government administrative unit). Each *kebele* is further divided into *gares*, with each *gare* having an average of 35 households. The geographic location of the district and the selected *kebeles* are shown in Fig. 1. The major rainy season is from June to August, whereas the minor rainy season is from February to March. Malaria is a major health problem, and the shores of Zeway Lake and the Bulbula River provide the main mosquito breeding sites [15]. Malaria transmission is seasonal, with the majority of cases occurring from September to December each year. There is one health post in each *kebele* staffed by two health extension workers, who provide comprehensive preventive services to the community, including malaria diagnosis and treatment.

### Study design and participants

A cohort of 5309 residents from 996 households in 29 *gares* selected from four *kebeles* was followed from August to December 2013. The study participants were selected through the following procedure: A preliminary mapping and census of 24 *kebeles* (sampling frame) in the Adami Tullu district was done in March and April 2013, with four *kebeles* selected from the enumerated 24 *kebeles* based on their distance from the main mosquito breeding sites (the Zeway lakeshore and the Bulbula River) and malaria burden (district health office malaria report). Two of the *kebeles*, Bochesa and Anano-Shisho, were located within 5 km and the other two, Elka-Chelemo and Gallo Raphe, which were beyond 5 km from the Zeway lake-shore and Bulbula River. To make access easy for patients with clinical sign of malaria, 29 *gares* within a 3-km radius from the community health posts were included. All the residents (5309) in these villages were invited to participate in the baseline survey in August 2013, and were followed-up weekly for 16 weeks.

### Weekly home visits and identification of malaria infection

Households were visited weekly (active surveillance), and household members with fever (body temperature ≥37.5 °C and/or history of fever in the last 48 h) were identified and referred to health posts. In addition, residents were asked to visit the health posts if they developed a fever when the project staff was not available (passive surveillance). Blood samples were collected by a finger prick of all febrile cases reported to the health posts, and rapid diagnostic tests (RDTs) and microscopic slide examination were performed. Patients with positive RDT were treated at the health posts with anti-malarial drugs (CoArtem^®^ for *P. falciparum* and chloroquine for *P. vivax*) according to the national malaria treatment guidelines [9]. Microscopic slides were prepared according to WHO guidelines [16], and the slides were read by two expert microscopists elsewhere (one in Adama City, the capital of the East Shewa Zone and the other in the Bulbula Health Centre, Adami Tullu district), and the agreement of the two was reported. Discordant slides were read for a third time by a senior microscopist. Data on exposure variables such as socio-economic and demographic variables, ownership and use of insecticidal treated nets (ITNs), whether IRS had been carried out during the past year, history of fever and malaria treatment in householders were all collected using interviewer-administered questionnaires. Information on ITN use was obtained from household heads’ response to a question whether anyone in the households had slept under a mosquito net the night before the survey. Data were collected by diploma graduates, while the questionnaires were modified from a malaria longitudinal study tool used in southern Ethiopia [5].

### Entomological data collection

The entomological study was conducted from June to October 2013, and a total of 36 *gares* (12 per *kebele*) from each of the three *kebeles* (Bochesa, Elka Chelemo and Gallo Raphe) were randomly selected for adult mosquito collections. Households were selected at random from the 36 *gares*, and indoor and outdoor mosquito collections were undertaken every month. For indoor host-seeking mosquito collections, the Center for Disease Control and Prevention light traps (CDC-LTs) were set in one house per *gare* and allowed to operate from 6:00 pm to 6:00 am. The traps were hung approximately 45 cm above the floor near the feet of occupants protected by LLINs. Indoor and outdoor resting mosquitoes were collected using standard pyrethrum spray catches (PSC) and artificial pit shelters from 6:00 am to 9:00 am, respectively. The PSC was carried out from one randomly selected house per *gare*, while pit shelter collections were performed from six pits per *kebele* from a total of 18 *gare*s.

### Processing of mosquito samples

Mosquitoes were identified to species by morphological characteristics using an identification key [17]. *Anopheles gambiae* sibling species identification was carried out through the use of the polymerase chain reaction (PCR) method [18]. The head and thorax of each mosquito was separated from the abdomen and tested for the presence of *P. falciparum* and *P. vivax* circumsporozoite protein (CSP) by direct enzyme-linked immunosorbent assay (ELISA) [19].

The sources of mosquito blood meals were determined by the direct ELISA procedure using human and bovine antibodies [20]. The abdomens of unfed *Anopheles* were dissected for parity using Detinova’s ovary tracheation method [21]. The parity rate (PR) was measured as the ratio of parous mosquitoes to the total mosquitoes dissected, whereas *Anopheles* mosquito longevity and PR were used to estimate mosquito life expectancy based on the formula proposed by Davidson [22]. Since there was no direct observation of the gonotrophic cycle (gc), the age estimation of *An. arabiensis* and *An. pharoensis* was made on a gc value of 3 days [23]. Because of the small number of the other *Anopheles* species, longevity was only estimated for *An. arabiensis* and *An. pharoensis*.

### Insecticide susceptibility

The larvae and pupae of mosquitoes were collected from different breeding sites, and were reared to adults in big cages. Insecticide susceptibility tests were conducted on two species, *An. arabiensis* and *An. pharoensis*. The test for the former species was done in August 2013, a time when the aquatic forms are abundant. In October and November of the same year the tests for the latter species were undertaken. The WHO tube test [24] was employed, and the insecticides included four pyrethroids (0.05 % deltamethrin, 0.75 % permethrin, 0.05 % alphacypermethrin and 0.05 % lambdacyhalothrin) and two carbamates (0.1 % bendiocarb and 0.1 % propoxur). The pyrethroids were impregnated in June/July 2013 and expired in June/July 2014, while bendiocarb was impregnated in May 2012 and expired in May 2015. The dates of impregnation and the expiry of propoxur were July 2013 and July 2016.

After the morphological identification of females [17], 2-3-day-old, non-blood fed mosquitoes were exposed to either insecticide-impregnated filter papers or oil-impregnated papers as controls for 1 h. Each replicate contained 20 mosquitoes, and for each insecticide and control, five to six replicates varying from 100 to 120 mosquitoes were tested. After transferring to holding tubes, mosquitoes were kept for 24 h in a ventilated and humid box free of insecticides. A favourable temperature and humidity were created by placing a damp towel on top of the box. Mortality counts were taken at the end of 24 h, and when control mortality was between 5 and 20 %, the percentage mortality of the experimental was corrected using Abbott’s formula. The susceptibility and resistance status of the two vector populations was determined based on the WHO criteria of percentage mortality rates [24].

### Outcome variable

Malaria episodes measured by the microscopic slide examination of blood samples for the presence or absence of the malaria parasite was the response variable. Adult mosquito host-seeking and resting density were the response variable for entomological study.

### Explanatory variables

The multilevel mixed effect model included covariates associated with malaria episode (grouped into individual, household and *kebele* factors). The individual factors were ITN use, age group (<5, 5–14, 15–24, and ≥25 years) and gender, whereas the educational status of the head of the household and the wealth index were household factors. The *kebele* factors included the distance from lakeshore or river (within or beyond 5 km).

### Statistical analyses

Data were entered, cleaned and analysed using IBM SPSS version 21(SPSS Inc., Chicago) and Stata 13 (STATA Corp, College Station, Texas). Descriptive statistics including percentages, mean and standard deviations, were used to summarize the data. A multilevel mixed effects Poisson regression model was fitted to measure associations between response and predictor variables. Malaria incidence (count data) was assumed to follow a Poisson distribution based on random and independent occurrence. Hence, a three-level mixed effects Poisson model with log link was considered to account for malaria episodes, with clustering according to individuals, households and *kebeles*. Wald Chi square test was used to check the fitted model against an intercept-only model. After bivariate analysis, those variables with P < 0.25 [25] and main factors were included in the multivariate analysis. The significance level was set at 0.05. The proportion of people using an ITN was calculated by dividing the number of household residents sleeping under an ITN the night before the visit divided by all individuals in the household.

A household wealth index was constructed using a principal component analysis (PCA) [26]. Fourteen variables were included: electricity, watch, radio, television, mobile telephone, separate kitchen, bike, animal cart, bank account or credit association, water source, latrine, window, materials used for a wall and the roof of the house. The first principal component represented 22.8 % of the variance in the sample with an eigenvalue of 3.2. Households were then ranked into three wealth categories (poor, medium, rich). ArcGIS 10.2 was used for an analysis of village distance from the lakeshore, as well as Stata version13 (StataCorp, Texas) software to calculate the incidence rate ratio (IRR) and to fit the multilevel mixed effects Poisson model.

Variations in adult mosquito host-seeking and resting density, both within and among the *gare*s and the study months, were analysed using a non-parametric Kruskal–Wallis test. The human blood index (HBI) and bovine blood index (BBI) were calculated based on WHO guidelines [27], and all statistical results were considered significant at P < 0.05.

### Ethical approval

The study protocol and informed consent forms were reviewed and approved by the Institutional Review Board of the College of Health Sciences at Addis Ababa University, Ethiopia and by the Regional Committee for Medical and Health Research (ref: 2013/986/REK Vest), Norway. The national ethical clearance was obtained from the Ethiopian Ministry of Science and Technology (ref: 3.10/446/06). Written permission to undertake the study was obtained from the Oromia Regional Health Bureau, the East Shewa Zone Health Department and the Adami Tullu District Health Offices. Local leaders, village leaders and community elders were also informed about the purposes of the study. Participation in the study was voluntary, and informed consent was obtained from each participant above the age of 18 years. For participants less than 18 years old, consent was obtained from parents or caretakers, while verbal consent was obtained from household heads before routine mosquito collections. Patients who were positive for malaria by RDT were treated at the health posts according to the national guidelines for malaria treatment.

## Results

### Epidemiological findings

#### Socio-demographics variables

A total of 5309 individuals in 996 households from four *kebeles* were registered at baseline. The number of households per *kebele* was 325 (32.6 %) in Anano Shisho, 263 (26.4 %) in Bochesa, 210 (21.1 %) in Gallo Raphe and 198 (19.9 %) in Elka Chelemo. Three households (eight individuals) were lost to follow up, with an average household size of 5.3 persons. Nearly half, or 49.9 % (2651) of them were women, and the median age was 15 (IQR = 7–28) years.

#### Malaria incidence and prevention practices

**Fig. 2:**
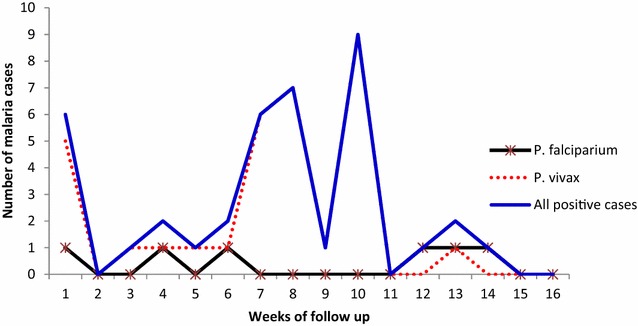
Malaria cases by *Plasmodium* species, Adami Tullu district, Ethiopia

Of 349 blood samples taken from febrile patients, 39 (11.2 %) slides were microscopically confirmed positive for malaria infection, and 12 (30 %) of these cases were identified through weekly home visits. *Plasmodium vivax* accounted for 33 (84.6 %) of the positive slides (Fig. 2), and patients with repeated malaria episodes were not observed. The overall malaria incidence was 4.6 cases per 10,000 person-weeks of observation (varied from 0 to 23.4 cases per 10,000 person-weeks of observation). However, the average malaria incidence for *gare*s close to the Zeway lakeshore and the Bulbula River was 7.8 per 10,000 person-weeks of observation. A high malaria incidence rate was observed in the under 5 years age group (6.8 episodes per 10,000 person-weeks) and from 5 to 14 years age group (6.4 episodes per 10,000 person-weeks) (Table 1).

**Table 1:** Socio-demographic variables and incidence rate of malaria among study participants, Adami Tullu district

Variables	Person-weeks of observation	Malaria cases	Per 10,000 person weeks
Age (years)			
Under 5	11,888	8	6.8
5–14	28,420	18	6.4
15–24	17,697	6	3.4
25 and above	26,819	7	2.6
Sex			
Male	42,523	22	5.2
Female	42,365	17	4.1
Distance from Lake /river			
≤5 km^a^	47,080	37	7.9
>5 km^b^	37,808	2	0.5
Wealth index			
Poor	27,024	6	2.2
Medium	28,778	11	3.8
Rich	28,910	22	7.7
HH head education			
No formal education	51,634	17	3.3
Primary	18,632	15	8.1
Secondary	8160	5	6.1
Above secondary	6462	2	3.1
ITN use			
No	82,569	38	4.6
Yes	2319	1	4.1

*HH* household

^a^ *kebeles* within 5 km (Bochesa and Anano Shisho)

^b^ *kebeles* beyond 5 km (Elika Chelemo and Gallo Rephe) from Zeway Lake shore and river (with potential mosquito breeding sites)

Less than one-third of the households (n = 271, 27.3 %) owned insecticide-treated nets. Data on ITN use (self-report of sleeping under ITNs last night) was collected during a census at the beginning of the study and once per week during the home visit. The percentage of ITN use among households that own an ITN was 49 % (n = 657), but the percentage for all participants was low (11 %). The median days individuals slept under an ITN for the 16 weekly visits was 2 days.

#### Determinants of malaria

The multivariate multilevel mixed effects model results (Table 2) showed that the age group from 5 to 14 years of age (IRR = 2.7; 95 % CI 1.1–6.6) was significantly associated with malaria episode. A high malaria episode (IRR = 2.82; 95 % CI 1.0–7.9) was also observed among children under 5 years of age, though due to a greater variance this was only borderline significant. Residents living in *kebeles* close to Zeway Lake and the Bulbula River had almost a 14.2 (95 % CI 3.1–64.7) times higher risk of malaria infection than those further away from the lake and river. The other variables, including gender, educational status of the head of the household, ITN use and wealth index, were not found to be predictors of malaria in the study area.

**Table 2:** Predictors of malaria episodes, Adami Tullu district

Variables	Fixed effects coefficients (IRR)	Standard error	95 % CI	P value
Sex				
Male	1.3	0.41	0.67–2.41	0.46
Female	1			
Age in years				
under 5	2.82	1.48	1.00–7.90	0.049*
5–14	2.7	1.23	1.1–6.60	0.028*
15–24	1.5	0.83	0.48–4.42	0.49
>24	1			
Distance from lake/river				
≤5 km	14.2	11	3.1–64.7	0.0001*
> 5 km	1			
Wealth status				
Poor	0.94	0.47	0.35–2.45	0.9
Medium	0.7	0.27	0.33–1.50	0.34
Rich	1			
HH head education				
No education	1.56	1.2	0.35–7.0	0.56
Primary	1.97	1.5	0.43–8.96	0.38
Secondary	1.67	1.43	0.31–9.0	0.55
Above secondary	1			
ITN use				
No	0.35	0.37	0.04–2.81	0.32
Yes	1			

*IRR* incidence rate ratio, *CI* 95 % confidence interval

* Significant at p < 0.05

### Entomological findings

#### *Anopheles* species composition and prevalence

**Fig. 3:**
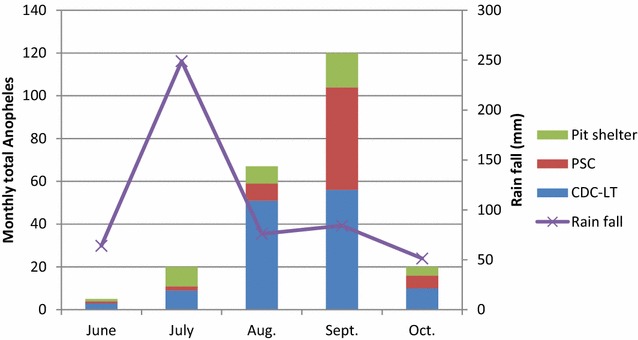
Monthly *Anopheles* abundance and average precipitation, Adami Tullu district, Ethiopia

Overall, 232 adult *Anopheles* mosquitoes were collected over the 5 months (Table 3). The species composition was 71.1 % *An. gambiae s.l.,* 21.1 % *An. pharoensis,* 5.2 % *An. ziemanni* and 2.6 % *An. funestus s.l*. All *An. gambiae* were confirmed to be *An. arabiensis* by PCR. The malaria vectors *An. arabiensis* and *An. pharoensis* occurred in all *kebeles,* with the largest proportion in Elka Chelemo (48.7 %) and the least in Gallo Raphe (18.5 %). The *Anopheles* abundance varied over the study months with a peak in September after the rainy season (Fig. 3). The average monthly precipitation peaked in July, and declined with a low precipitation from August to October.

**Table 3:** Species composition of adult *Anopheles* mosquitoes collected in Adami Tullu district

Village	Species	Collection method			Total
		CDC-LT	PSC	Pit shelter	
Bochesa	*An. arabiensis*	19 (14.7)	6 (9.2)	19 (50.0)	44 (19.0)
	*An. pharoensis*	12 (9.3)	2 (3.1)	0	14 (6.0)
Elka Chelemo	*An. arabiensis*	38 (29.5)	33 (50.8)	9 (23.7)	80 (34.5)
	*An. pharoensis*	30 (23.3)	3 (4.6)	0	33 (14.2)
	*An. funestus*	5 (3.9)	0	1 (2.6)	6 (2.6)
	*An. zeimanni*	9 (7.0)	0	3 (7.9)	12 (5.2)
Gallo Raphe	*An. arabiensis*	14 (10.9)	21 (32.3)	6 (15.8)	41 (17.7)
	*An. pharoensis*	2 (1.6)	0	0	2 (0.9)
Total	*Anopheles*	129 (55.6)	65 (28.0)	38 (16.4)	232

*CDC*-*LT* Center for Disease Control and Prevention light Trap, *PSC* Pyrethrum Spray Collection, figures in parentheses indicate percentage

#### Host-seeking and resting behaviour

**Fig. 4:**
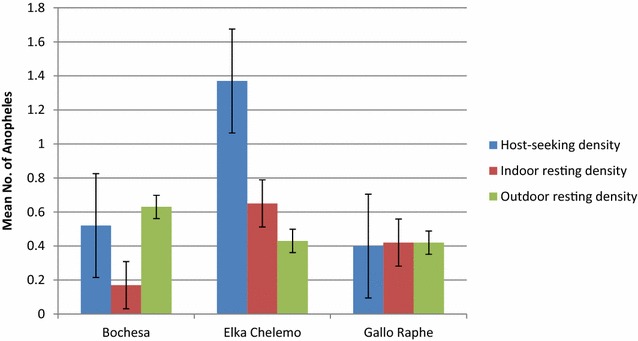
Mean indoor and outdoor density of *Anopheles,* Adami Tullu district, EthiopiaHost-seeking density (CDC-LT) = Mean no. Anopheles/light trap/night/house, indoor resting density (*PSC* pyrethrum spray catch) = mean no. *Anopheles*/house/45 min in a day, outdoor resting density (Pit shelter) = mean no. *Anopheles*/pit/30 min in a day

**Fig. 5:**
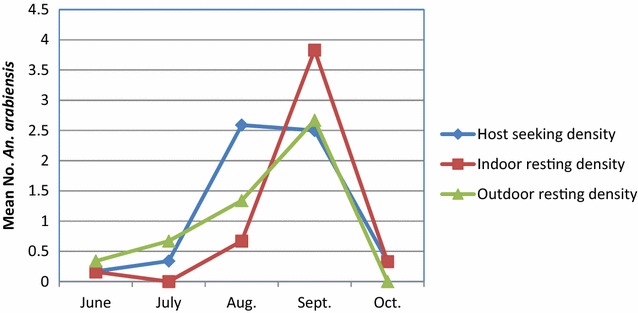
Overall average monthly host-seeking and resting densities of *An. arabiensis*, Adami Tullu district, Ethiopia. Host-seeking density (CDC-LT), indoor resting density (*PSC* pyrethrum spray catch), outdoor resting density (Pit shelter)

The mean host-seeking density of *Anopheles* collected by CDC-LT indoors was 0.7 *Anopheles* per CDC-LT/night/house. The mean indoor resting density of *Anopheles* obtained by PSC was 0.4 *Anopheles* per house per day, whereas the mean outdoor resting density collected from pit shelters was 0.4 *Anopheles* per pit shelter per day over the 5 months. The highest mosquito density was found in Elka Chelemo, where there were significant differences between collection methods (Fig. 4). The average indoor host-seeking density, indoor resting density and outdoor resting density of *An. arabiensis* generally peaked in September, and almost declined to zero in October (Fig. 5).

*Anopheles* abundance varied significantly between *kebeles* and *gares*. There were significant differences in the abundance of anopheline mosquitoes between the three *kebeles* (Kruskal–Wallis test = 11.25, df = 2, P = 0.004) and between the 36 *gares* (Kruskal–Wallis test = 68.93, df = 35, P = 0.001). The same statistical test revealed that there were significant differences in host-seeking abundances (light trap catches) of *An. arabiensis* (P = 0.025), *An. pharoensis* (P = 0.001) and *An. zeimanni* (P = 0.015) between *kebeles*. However, the indoor host-seeking abundance (light traps) of *An. funestus* s.l. was not significantly different between *kebeles* (P = 0.458).

Moreover, no significant differences were detected between *kebeles* and *gares* in the abundance of indoor resting anophelines (PSC) (P > 0.05) and outdoor resting anophelines (pit shelter) (P > 0.05).

#### Blood meal sources of *Anopheles* mosquitoes

Of 107 freshly fed *Anopheles* tested, the overall HBI and BBI was 0.70 and 0.38, respectively (Table 4), with the overall HBI and BBI for *An. arabiensis* 0.69 and 0.39, respectively. *Anopheles arabiensis* preferred to feed more on humans (0.59) than bovines (0.29). The HBI was higher for *An. arabiensis* collected indoors (0.79) than for those collected outdoors (0.37). Inversely, the BBI was higher for *An. arabiensis* caught outdoors (0.68) compared to those collected indoors (0.27). All *An. pharoensis* females that had fed on humans were captured indoors, though none of the indoor- and outdoor-collected *An. pharoensis* females had taken blood from bovines alone. *Anopheles ziemanni* fed more on bovine (BBI = 0.67) than human (HBI = 0.50).

**Table 4:** Blood meal sources of the *Anopheles* species, Adami Tullu district

*Anopheles* species	Collection venues	No. analyzed N (HBI)	Blood meals sources			
			Human N (HBI)	Bovine N (BBI)	Mixed N (MBI)	Unknown N
*An. arabiensis*	CDC-LT	24 (0.75)	15 (0.63)	6 (0.25)	3 (0.13)	0
	PSC	48 (0.79)	35 (0.73)	10 (0.21)	3 (0.06)	0
	Pit shelter	19 (0.37)	4 (0.21)	10 (0.53)	3 (0.16)	2 (0.11)
	Total	91 (0.69)	54 (0.59)	26 (0.29)	9 (0.09)	2 (0.02)
*An. pharoensis*	CDC-LT	7 (1.00)	6 (0.86)	0	1 (0.14)	0
	PSC	2 (1.00)	2 (1.00)	0	0	0
	Total	9 (1.00)	8 (0.89)	0	1 (0.11)	0
*An. ziemanni*	CDC-LT	3 (0.67)	2 (0.67)	1 (0.33)	0	0
	Pit shelter	3 (0.33)	0	2 (0.67)	1 (0.33)	0
	Total	6 (0.50)	2 (0.33)	3 (0.50)	1 (0.17)	0
*An. funestus*	Pit shelter	1	0	1	0	0
Overall *Anopheles*		107 (0.7)	64 (0.60)	30 (0.30)	11 (0.10)	2 (0.20)

When computing for human blood index (*HBI*) and bovine blood index (*BBI*), mixed blood meals were added to the number of human blood and bovine blood meals. Mixed blood meals = human + bovine, unknown blood meals are negative for both human and bovine antibodies, show Soverall HBI

#### Sporozoite rate, parity rate and longevity of the malaria vectors

All collected mosquitoes (n = 232) were negative for *P. falciparum* and *P. vivax* circumsporozoite proteins. Table 5 shows the parity rate and average longevity of *An. arabiensis* and *An. pharoensis*. The overall average age of *An. arabiensis* and *An. pharoensis* females was 14 days (range: 7–25 days) and 1.6 days (range: 0–6.3 days), respectively.

**Table 5:** Parity rates and longevity of *Anopheles* species, Adami Tullu district

Kebeles	Species	Number of mosquitoes					
		Collected	Dissected	Parous	PR	P	Age (Days)
Bochesa	*An. arabiensis*	44	3	2	0.67	0.87	7
	*An. pharoensis*	14	5	1	0.20	0.58	1.8
Elka Chelemo	*An. arabiensis*	80	9	8	0.89	0.96	25
	*An. pharoensis*	33	18	11	0.61	0.85	6.3
Gallo Raphe	*An. arabiensis*	41	3	2	0.67	0.87	7
	*An. pharoensis*	2	2	0	0.00	0.00	0
Average for *An. arabiensis*		55	5	4	0.8	0.93	14
Average for *An. pharoensis*		16.33	25	4	0.16	0.54	1.6

*PR* parity rate, *P* probability of surviving 1 day

#### Indoor host-seeking density of *Anopheles* and malaria episodes

**Fig. 6:**
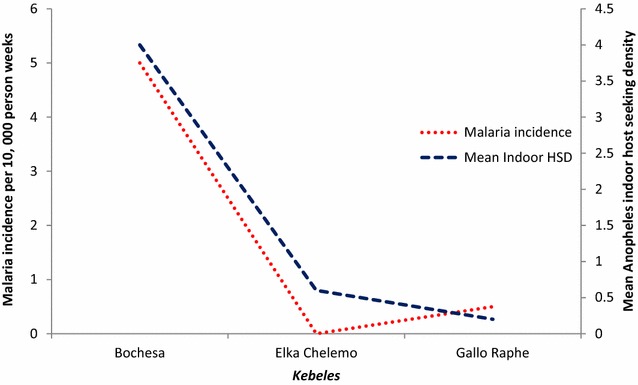
Malaria incidence and mean indoor host seeking density of *Anopheles*, Adami Tullu district, Ethiopia

Both epidemiological and entomological collection was done in 13 *gares* from three *kebeles* (Bochesa, Elka Chelemo and Gallo Raphe). A higher mean (four) indoor host-seeking density of *Anopheles* mosquito was observed in *kebele* (Bochesa) near a lake. In the same *kebele*, a higher (five episodes per 10,000 person week) malaria incidence was observed (Fig. 6).

#### Status of insecticide susceptibility of *An. arabiensis* and *An. pharoensis*

**Fig. 7:**
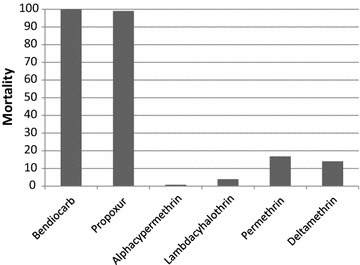
Insecticide susceptibility of *An. arabiensis,* Adami Tullu district, Ethiopia

**Fig. 8:**
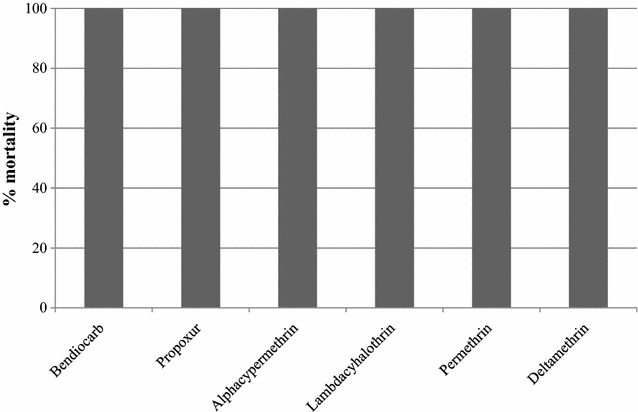
Insecticide susceptibility of *An. pharoensis,* Adami Tullu district, Ethiopia

*Anopheles arabiensis* was highly resistant to deltamethrin, lambdacyhalothrin, permethrin and alphacypermethrin (mortality 0.8–16.8 %), but susceptible to bendiocarb and propoxur (Figs. 7, 8). All tested *An. pharoensis* were found susceptible to all insecticides, as mortality in all cases was 100 %. Control mortality varied from 0–16 % (higher mortality was encountered during the permethrin tests).

## Discussion

This study represents one of the few descriptions from Ethiopia on simultaneous epidemiological and entomological information. The overall malaria incidence rate was 4.6 cases per 10,000 person-weeks of observation. However, the incidence varied between the *gares*, and in *gares* closer to the lake or river, the incidence rate was close to eight cases per 10,000 person-weeks of observation. *Plasmodium vivax* was the dominant species, and a higher malaria incidence was observed in a *kebele* with overall higher mean host-seeking density of *Anopheles*.

The observed malaria incidence in this study was higher (4.6 versus 3.6 episodes per person weeks) than what has been reported from southern Ethiopia [5], which could be due to the short (16 versus 101 weeks) follow-up period in the current study.

In line with previous studies from other parts of the country [28, 29], *P. vivax* was the dominant species in the study area, representing about 85 % of the positive cases. The 2011 malaria indicator survey also reported *P. vivax* as the main (60 %) causative agent in the Oromia region [3].

Although all of the study *kebeles* were located below 2000 metres above sea level, which is defined as the threshold for being a malarious area, differences in malaria distribution were observed among the *kebeles* and *gares*. Those *Kebeles* near to the lake or river had more incidences of malaria and a higher mean indoor host-seeking density of *Anopheles* mosquito, with similar observations done in other parts of Ethiopia [30]. The persistent presence of infectious *Anopheles* mosquitoes and of water bodies [31] could have resulted in high number of malaria cases in the lakeshore and river areas.

Comparable to other studies [5], children in the age group of under 5s and from 5 to 14-year were more likely to develop malaria than older people. It has been reported that children are more at risk of developing malaria than adults in lowland areas (area <2000 metres above sea level) or malaria-endemic areas [32]. It is a well-known fact that children have a lower immunity to malaria than adults [33].

Families’ welfare, as measured by the wealth index, was not associated with the probability of malaria episodes in this study. The inclusion of villages close to a health post (within 3 km), as well as weekly home visits to identify residents with clinical symptoms of malaria, could have motivated febrile cases to seek early treatment and reduce the risk of transmission to other family members in both poor and rich families. The ownership of ITNs in this study (27.3 %) was much lower than the WHO recommendations and the national goal (100 % coverage) [1, 8, 34], and also lower than what is reported from other parts of the country [35].

*Anopheles arabiensis* and *An. pharoensis* were the predominant species in Adami Tullu *kebeles.* These findings were consistent with Abose et al. [36], who reported *An.arabiensis* as the primary- and *An. pharoensis* as secondary vectors in the area. The results also showed that the monthly average precipitation peaked in July and sharply declined from August to October, whereas *Anophele*s abundance rose in September and sharply dropped in October. This was expected since *Anopheles* population dynamics and malaria transmissions are driven by seasonal precipitation in Ethiopia [37]. *Anophelesarabiensis* proliferates in rain-fed residual pools after months of heavy rain in the country [7] and the populations expand during this time; however, excessive rainfall may flush out breeding pools [38]. Therefore, the peak *Anopheles* abundance may not coincide with peak precipitation months.

Results indicate that the overall mosquito density captured by the different mosquito sampling methods was low compared to previous studies in the area [36]. The reason for the low *Anopheles* density could be the rapid scale-up and intensive use of vector intervention measures, particularly ITNs and IRS in the country [28] and elsewhere in eastern Africa [39]. Besides, global climatic changes, particularly changes in hydrologic and climatic factors such as precipitation, humidity, temperature and wind [40], may have adversely impacted the *Anopheles* population controlling breeding and survival.

The other key potential reason for the low mosquito catches could be the lack of efficient mosquito sampling tools [39]. Efficient indoor and outdoor collection tools are required, especially for vectors such as *An. arabiensis,* which have behavioural plasticity in host preferences and shifts in peak biting time [41]; hence, there is a need to address the inefficient catching techniques. Because adult mosquitoes occur at a certain radius from their breeding sites, a district-wide random sampling of adult mosquitoes without referring to any mosquito breeding sites could also have a potential impact on the occurrence and abundance of mosquitoes, and needs to be revisited.

The overall HBI (0.69) for *An. arabiensis* was higher than the BBI (0.38) for the same species. This finding contrasts prior studies that found a higher BBI for *An. arabiensis* than the HBI in the country [4]. However, the present finding is in line with [42], which found a higher HBI for *An. arabiensis* compared to the BBI in the country. It should be noted that the present study used similar mosquito sampling methods than the previous study [4], thus the potential influence of mosquito trapping on HBI is not expected. But the present finding was similar to the other study [42], that relied on the CDC light trap alone for mosquito collection, which is evidence that the trapping methods used did not impact the HBI. The HBI for *An. arabiensis* was higher indoors (0.73) than outdoors (0.21), but the BBI was higher when collected outdoors (0.53) than indoors (0.21). These results are generally in agreement with prior studies, which observed the opportunistic feeding behaviour of *An. arabiensis* [43]. *Anopheles pharoensis* showed anthropophilic and endophilic behaviour in the area, but more blood-fed females should be tested to reach such conclusions. Furthermore, the average longevity of *An. arabiensis* ranged from 7 to 25 days in the villages, thereby implying that the vector had a sufficient longevity for malaria transmission during the study period.

Overall, the relative increase in the abundance of mosquitoes in September and the beginning of October, compared to the other study months, coincides with an increased incidence of malaria episodes in the same and subsequent months. In the entomological study, an overall high mosquito density was observed in Elka Chelemo kebele, although the malaria incidence for the *kebele* was low. This could be due to some of the *gares* being located near the lakeshore in this *kebele,* where a higher mosquito abundance was not followed for malaria episodes. In *kebeles* where the two studies overlapped, high malaria episodes were observed in those with a higher indoor host-seeking density of *Anopheles.*

*Anopheles arabiensis* was highly resistant to all the tested pyrethroids, including deltamethrin, but susceptible to bendiocarb and propoxur. The insecticide susceptibility study showed a resistance of *An. arabiensis* to the pyrethroids, which is the current insecticide of choice for the treatment of LLINs. This resistance may compromise the efficacy and effectiveness of ITNs. On the other hand, *An. arabiensis* is currently susceptible to the carbamates, which is useful and will be beneficial for the main trial.

The use of weekly active case detection, which was supplemented with passive surveillance, would have maximized the number of cases captured by the study. In addition, the active monitoring of the occurrence and the abundance of adult mosquitoes through house-to-house surveys during the major malaria transmission season in the area is also another strength. Weaknesses of the study include a lack of efficient mosquito sampling methods for the local vectors, the overestimation of malaria incidence due to the inclusion of the major malaria transmission season and the self-report of sleeping under ITNs (difficult to observe), which may have affected the reported ITN usage.

## Conclusions

This pilot study showed a high variation of malaria incidence and *Anopheles* among *gares*. Younger age groups and households located near lakes and rivers were significantly associated with malaria infection. *Anopheles arabiensis* had a high HBI (high human contact) and a sufficient longevity for malaria transmission. The observed susceptibility of the malaria vectors to propoxur warrants using this insecticide for indoor residual spraying, and results from this study will be used as a baseline for the trial.
